# Association between changes in working status and hand-grip strength among Korean middle-aged and older adults: a longitudinal panel study

**DOI:** 10.1038/s41598-022-16373-2

**Published:** 2022-07-28

**Authors:** Il Yun, Yu Shin Park, Eun-Cheol Park, Sung-In Jang

**Affiliations:** 1 Department of Public Health, Graduate School, Yonsei University, Seoul, Republic of Korea; 2 Institute of Health Services Research, Yonsei University, Seoul, Republic of Korea; 3 Department of Preventive Medicine, Yonsei University College of Medicine, 50-1 Yonsei-to, Seodaemun-gu, Seoul, 03722 Republic of Korea

**Keywords:** Diseases, Health care

## Abstract

We investigated the association between working status changes and hand-grip strength (HGS) among middle-aged and older Korean adults using data from the 2006–2018 Korean Longitudinal Study of Aging. After excluding those with less than normal HGS in the baseline year, newly added panels, and missing values, 3843 participants (2106 men; 1737 women) were finally included. After adjusting for potential confounders, we used a 2-year lagged multivariable generalized estimating equation model to examine this association longitudinally. Men who quit working or who continued to be non-working were more likely to have lower HGS than those who continued to work (working → non-working, adjusted odds ratio [OR]: 1.47, 95% confidence interval [CI] 1.26–1.70; non-working → non-working, adjusted OR: 1.52, 95% CI 1.34–1.72). Compared to women who continued to work, the other three groups showed high ORs with low HGS (working → non-working, adjusted OR: 1.19, 95% CI 1.01–1.40; non-working → working, adjusted OR: 1.18, 95% CI 0.98–1.42; non-working → non-working, adjusted OR: 1.38, 95% CI 1.22–1.56). Middle-aged and older adults whose working status changed to non-working were at higher risk of reduced HGS than others and required muscular strength training interventions to improve HGS and prevent sarcopenia.

## Introduction

The loss of muscle mass and strength is a prominent feature of the aging population and is commonly characterized by sarcopenia^1,2^. Studies on sarcopenia have increased, revealing that muscle strength reduction results in poor health outcomes such as functional disabilities, cognitive decline, chronic morbidities, and all-cause mortality^3–6^. Hand-grip strength (HGS)—a simple, quick, reliable, and inexpensive method for measuring strength—is used for measuring the overall muscle strength in older adults and is known to be correlated with leg strength^7,8^. Recent studies have proved the validity of using hand dynamometers to assess health and nutritional status^9,10^, and several previous studies have used HGS as a proxy measure for muscle function and physical health^2,11–13^. HGS measurement is increasingly common as a clinically viable screening tool for determining muscle weakness and detecting other clinically relevant health outcomes^1^. Both the Asian Working Group for Sarcopenia (AWGS) and the European Working Group on Sarcopenia in Older People (EWGSOP) also apply HGS as a criterion for sarcopenia^7,8^.

Unemployment is known to affect health adversely^14^. Previous studies have shown that unemployed older people have poor physical and mental health^15,16^ and low quality of life^17^, proving that economic activity in old age is an important determinant of health and quality of life. Furthermore, older people change their working status more frequently than young people for various reasons, such as retirement and health problems, leading to diverse health outcomes. There are many studies on the health status of older people who quit economic activity^18,19^. Still, few studies have attempted to examine the effects of changes in working status in old age on health outcomes using longitudinal data. Moreover, when investigating the relationship between working status and health, HGS has rarely been used to indicate overall strength and health in older people.

Therefore, we aimed to investigate the association between changes in working status and HGS among Korean middle-aged and older adults based on a longitudinal panel study. Confirming this association through a large sample of population-based panel data would help establish a basis for improving the health and well-being of the aging population.

## Methods

### Data and study population

The data analyzed in this study were obtained from the Korean Longitudinal Study of Aging (KLoSA). The KLoSA is a longitudinal panel survey of nationally representative Koreans aged ≥ 45 years, excluding institutionalized people and Jeju Island residents. The Korea Labor Institute has conducted a survey every 2 years since 2008 to build primary data for establishing effective social and welfare policies and preparing for an aging society. Data pertaining to family, health, employment, income, assets, subjective expectations, and quality of life are collected^20,21^. As the KLoSA is publicly accessible and informed consent was obtained from all participants, no further ethical approval was required.

**Figure 1:**
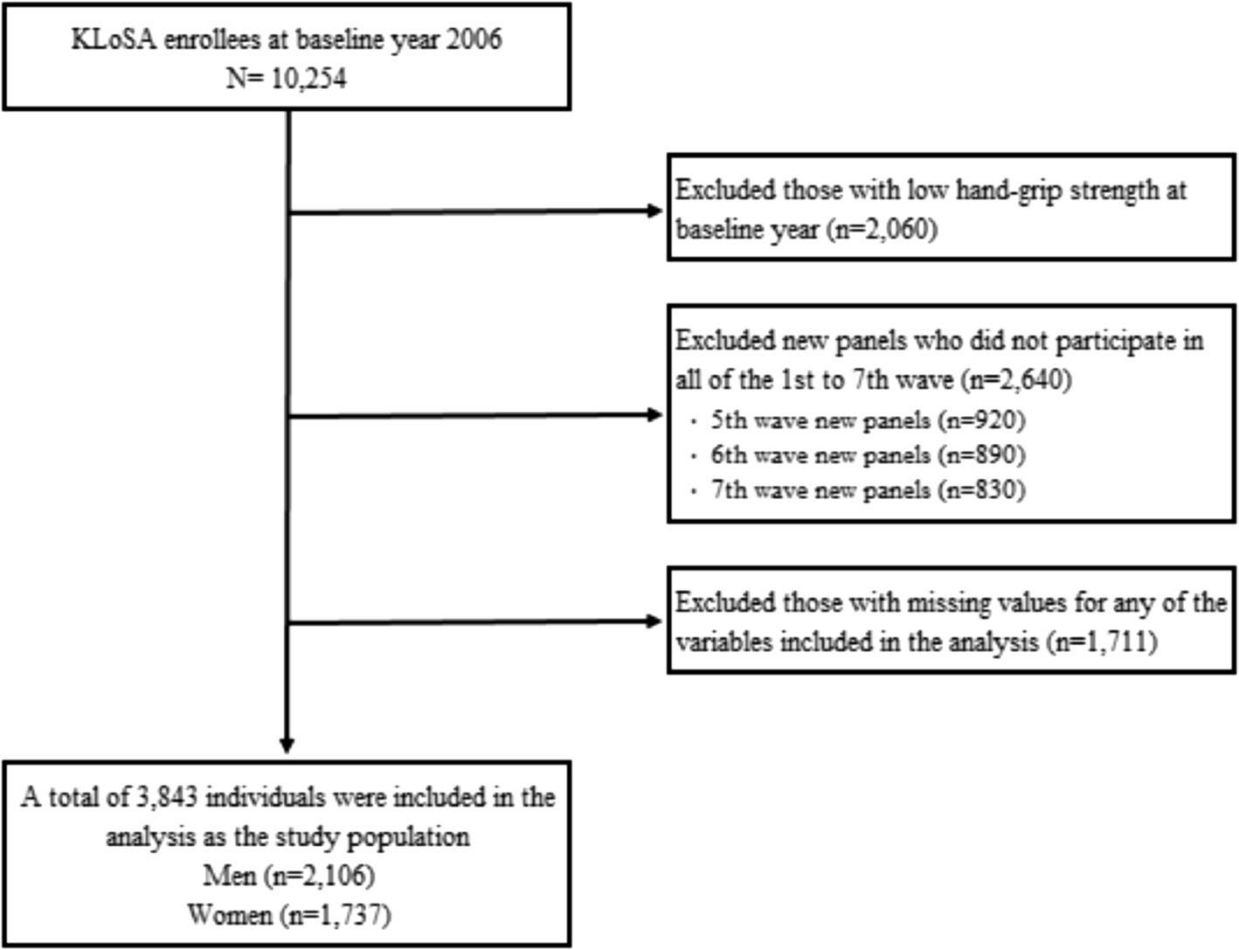
Flow chart of sample selection.

In this study, we utilized data from the first to seventh waves of the KLoSA (2006–2018). In all, 10,254 individuals participated in the baseline survey conducted in 2006. Considering the study hypothesis, those with low HGS in the baseline year were excluded (n = 2060). In addition, individuals that did not participate in the first to seventh waves (n = 2640) and those with missing values (n = 1711) were excluded. Finally, 3843 individuals (2106 men; 1737 women) were included in the analysis as the study population. In conducting this observational study, we followed the STROBE checklist^22^. The detailed flow of the sample selection process is depicted in Fig. 1.

### Measures

HGS, the dependent variable in this study, was measured in kilograms using a hand-grip dynamometer (Model number: 6103, TANITA, Japan). The participants in the KLoSA were asked to squeeze the dynamometer twice for each hand in a sitting position, and the mean value of four trials was recorded. Since previous studies have confirmed that HGS is significantly different in men and women^13,23^, we analyzed the data by stratification according to sex. In the present study, HGS was analyzed as a categorical variable according to the criteria presented by the AWGS in 2019. Low HGS was defined as those with HGS < 28 kg for men and < 18 kg for women^24^.

Each participant was asked about their working status through the question, "Are you currently working for income?" In this context, working included being employed, running their own business, and helping a family member or relatives. The response options were "Yes" or "No". Changes in working status per wave, which was the main variables of interest, were classified into four groups: (1) working → working, (2) working → non-working, (3) non-working → working, and (4) non-working → non-working.

Covariates included socioeconomic and health-related variables for each wave. Socioeconomic factors included age (45–59, 60–69, 70–79, and ≥ 80 years), marital status (married, unmarried, or separate), region (urban and rural), the highest level of education (middle school and below, high school, and college and above), and personal income level, which were divided into tertiles, with tertile 3 indicating the highest earner. Health-related factors included satisfaction with health status, body mass index (BMI), activities of daily living (ADL), instrumental activities of daily living (IADL), cognitive function, and regular physical exercise. Perceived satisfaction with health status was classified into dissatisfied, average, and satisfied. BMI was calculated from self-reported height and weight data on the first wave and divided into three groups (underweight: BMI < 18.5 kg/m^2^, normal: BMI 18.5–24.95 kg/m^2^, and overweight: BMI ≥ 25 kg/m^2^). ADL and IADL indices were classified as 0 and 1, with 0 points indicating normality and 1 point indicating the need for help^25^. Cognitive function was measured by the Korean version of the MMSE score during the first wave, and with a total score of 30, the cut-off level for cognitive impairment was 24 points^26^. Regular physical exercise was defined as "Yes" if they exercised at least once a week and "No" if not^25^.

### Statistical analysis

The chi-square test was conducted to investigate and compare the general characteristics of the study population. We also used a 2-year lagged multivariable generalized estimating equation (GEE) model to longitudinally examine the association between changes in working status and HGS after adjusting for potential confounders^27^. The key results are presented as odds ratios (ORs), regression coefficients (β), and 95% confidence intervals (CIs). The statistical package SAS version 9.4 (SAS Institute Inc.; Cary, NC, USA) was used for all analyses, and p-values < 0.05 were deemed statistically significant.

## Results

Table 1 shows the general characteristics of the study population in the baseline year (2006–2008) at the first time point of changes in working status. The four groups of changes in working status showed statistically significant differences in HGS for both sexes. Other covariates also showed significant differences in HGS for men, except for region, and women, except for region and BMI.

**Table 1:** General characteristics of the study population (2006 → 2008 baseline year).

Variables	Men (N = 2106)							Women (N = 1737)						
	Hand-grip strength (HGS)^a^							Hand-grip strength (HGS)^a^						
	Total		Normal		Low		*P-value*	Total		Normal		Low		*P-value*
	N	%	N	%	N	%		N	%	N	%	N	%	
**Changes in working status**							< 0.0001							< 0.0001
Working → working	1412	67.0	1243	88.0	169	12.0		610	29.0	530	86.9	80	13.1	
Working → non-working	124	5.9	94	75.8	30	24.2		87	4.1	72	82.8	15	17.2	
Non-working → working	84	4.0	71	84.5	13	15.5		144	6.8	112	77.8	32	22.2	
Non-working → non-working	486	23.1	332	68.3	154	31.7		896	42.5	621	69.3	275	30.7	
**Age**							< 0.0001							< 0.0001
45–59	1062	50.4	978	92.1	84	7.9		787	37.4	711	90.3	76	9.7	
60–69	658	31.2	533	81.0	125	19.0		590	28.0	440	74.6	150	25.4	
70–79	332	15.8	204	61.4	128	38.6		321	15.2	171	53.3	150	46.7	
80 or older	54	2.6	25	46.3	29	53.7		39	1.9	13	33.3	26	66.7	
**Marital status**							0.032							< 0.0001
Married	1969	93.5	1636	83.1	333	16.9		1248	59.3	996	79.8	252	20.2	
Unmarried or being separately	137	6.5	104	75.9	33	24.1		489	23.2	339	69.3	150	30.7	
**Region**							0.434							0.906
Urban	925	43.9	771	83.4	154	16.6		769	36.5	590	76.7	179	23.3	
Rural	1181	56.1	969	82.0	212	18.0		968	46.0	745	77.0	223	23.0	
**Highest level of education**							< 0.0001							< 0.0001
Middle school or below	880	41.8	659	74.9	221	25.1		1272	60.4	936	73.6	336	26.4	
High school	823	39.1	725	88.1	98	11.9		390	18.5	331	84.9	59	15.1	
College and above	403	19.1	356	88.3	47	11.7		75	3.6	68	90.7	7	9.3	
**Personal income level**							< 0.0001							< 0.0001
Tertile 1 (low)	671	31.9	479	71.4	192	28.6		579	27.5	424	73.2	155	26.8	
Tertile 2	721	34.2	618	85.7	103	14.3		598	28.4	433	72.4	165	27.6	
Tertile 3 (high)	714	33.9	643	90.1	71	9.9		560	26.6	478	85.4	82	14.6	
**Satisfaction with health status**							< 0.0001							< 0.0001
Dissatisfied	189	9.0	131	69.3	58	30.7		241	11.4	151	62.7	90	37.3	
Average	1265	60.1	1018	80.5	247	19.5		1143	54.3	889	77.8	254	22.2	
Satisfied	652	31.0	591	90.6	61	9.4		353	16.8	295	83.6	58	16.4	
**BMI**^**b**^							0.001							0.440
Underweight	34	1.6	21	61.8	13	38.2		41	1.9	28	68.3	13	31.7	
Normal	1620	76.9	1323	81.7	297	18.3		1250	59.4	962	77.0	288	23.0	
Overweight	452	21.5	396	87.6	56	12.4		446	21.2	345	77.4	101	22.6	
**ADL Index**^**c**^							0.006							0.001
0 (Normal)	2096	99.5	1735	82.8	361	17.2		1724	81.9	1330	77.1	394	22.9	
1 (Need help)	10	0.5	5	50.0	5	50.0		13	0.6	5	38.5	8	61.5	
**IADL Index**^**c**^							0.001							< 0.0001
0 (Normal)	1904	90.4	1590	83.5	314	16.5		1677	79.6	1302	77.6	375	22.4	
1 (Need help)	202	9.6	150	74.3	52	25.7		60	2.8	33	55.0	27	45.0	
**Cognitive function**^**d**^							< 0.0001							< 0.0001
Normal	1916	91.0	1627	84.9	289	15.1		1335	63.4	1115	83.5	220	16.5	
Impairment	190	9.0	113	59.5	77	40.5		402	19.1	220	54.7	182	45.3	
**Regular physical exercise**							< 0.0001							0.080
Yes	893	42.4	773	86.6	120	13.4		630	29.9	499	79.2	131	20.8	
No	1213	57.6	967	79.7	246	20.3		1107	52.6	836	75.5	271	24.5	

BMI, Body Mass Index; ADL, Activities of Daily Living; IADL,Instrumental Activities of Daily Living.

^a^As defined by AWGS (Asian Working Group for Sarcopenia), cut-off points of hand-grip strength were 28 kg for men and 18 kg for women.

^b^Underweight (BMI of less than 18.5 kg/m^2^), Normal (BMI of 18.5–24.9 kg/m^2^), Overweight (BMI of 25 kg/m^2^ or over).

^c^0 point (If they do not need any help), 1 point (If they need some help, or if they need help altogether).

^d^Normal (MMSE score of 24 points or more), Impairment (MMSE score of 23 points or less).

Table 2 presents the results of the GEE analysis of the association between changes in working status and HGS after adjusting for all potential confounding variables. Among men, it was found that those who quit working and those who continued to be non-working were more likely to have lower HGS than those who continued to work (working → non-working, adjusted OR: 1.47, 95% CI 1.26–1.70; non-working → non-working, adjusted OR: 1.52, 95% CI 1.34–1.72). Conversely, in women, compared to those who continued to work, all of the other three groups showed high ORs with low HGS (working → non-working, adjusted OR: 1.19, 95% CI 1.01–1.40; non-working → working, adjusted OR: 1.18, 95% CI 0.98–1.42; non-working → non-working, adjusted OR: 1.38, 95% CI 1.22–1.56). A similar tendency was also observed in analyzing HGS as a continuous variable. Compared with the group who continued to work, the group who quit working and those who continued to be non-working showed highly significant regression coefficients (Supplementary Table 1).

**Table 2:** Results of GEE analysis of factors associated with low hand-grip strength.

Variables	Men		Women	
	HGS < 28kg^a^		HGS < 18kg^a^	
	Adjusted OR	95% CI	Adjusted OR	95% CI
**Changes in working status**				
Working → working	1.00		1.00	
Working → non-working	1.47	(1.26–1.70)	1.19	(1.01–1.40)
Non-working → working	0.97	(0.77–1.21)	1.18	(0.98–1.42)
Non-working → non-working	1.52	(1.34–1.72)	1.38	(1.22–1.56)
**Age**				
45–59 years	1.00		1.00	
60–69 years	1.58	(1.38–1.81)	1.84	(1.62–2.09)
70–79 years	2.91	(2.48–3.40)	3.51	(3.03–4.07)
80 years or older	7.93	(6.40–9.83)	6.89	(5.60–8.48)
**Marital status**				
Married	1.00		1.00	
Unmarried or Being separately	0.99	(0.83–1.18)	1.17	(1.05–1.29)
**Region**				
Urban	1.00		1.00	
Rural	0.90	(0.82–1.00)	0.87	(0.78–0.95)
**Highest level of education**				
Middle school or below	1.27	(1.09–1.37)	1.68	(1.29–2.19)
High school	1.01	(0.86–1.18)	1.38	(1.05–1.82)
College and above	1.00		1.00	
**Personal income level**				
Tertile 1 (low)	1.34	(1.18–1.53)	1.20	(1.07–1.34)
Tertile 2	1.14	(1.01–1.28)	1.22	(1.10–1.35)
Tertile 3 (high)	1.00		1.00	
**Satisfaction with health status**				
Dissatisfied	1.67	(1.41–1.97)	1.89	(1.63–2.18)
Average	1.20	(1.08–1.34)	1.22	(1.09–1.37)
Satisfied	1.00		1.00	
**BMI**^**b**^				
Underweight	1.85	(1.43–2.41)	1.64	(1.29–2.09)
Normal	1.00		1.00	
Overweight	0.70	(0.62–0.79)	0.85	(0.77–0.94)
**ADL Index**^**c**^				
0 (Normal)	1.00		1.00	
1 (Need help)	2.19	(1.44–3.33)	1.32	(0.94–1.86)
**IADL Index**^**c**^				
0 (Normal)	1.00		1.00	
1 (Need help)	0.79	(0.68–0.92)	1.23	(1.02–1.49)
**Cognitive function**^**d**^				
Normal	1.00		1.00	
Impairment	1.79	(1.58–2.01)	1.94	(1.76–2.13)
**Regular physical exercise**				
Yes	1.00		1.00	
No	1.35	(1.23–1.49)	1.05	(0.96–1.15)

*GEE* generalized estimating equation, *OR* odds ratio, *CI* confidence interval, *HGS* hand-grip strength, *BMI* Body Mass Index, *ADL* Activities of Daily Living, *IADL* Instrumental Activities of Daily Living.

^a^Criteria defined by AWGS (Asian Working Group for Sarcopenia) in 2019.

^b^Underweight (BMI of less than 18.5 kg/m^2^), Normal (BMI of 18.5–24.9 kg/m^2^), Overweight (BMI of 25 kg/m^2^ or over).

^c^0 point (If they do not need any help), 1 point (If they need some help, or if they need help altogether).

^d^Normal (MMSE score of 24 points or more), Impairment (MMSE score of 23 points or less).

Additionally, we conducted sensitivity analyses to examine this association using different criteria for defining HGS (Table 3). The European Working Group on Sarcopenia in Older People 2 defined the criteria for low HGS as < 27 kg for men and < 16 kg for women^28,29^. We also performed the analysis by defining the low 25 percentiles of KLoSA, the data used in this study, as low HGS. The criteria were < 26.75 kg for men and < 16.25 kg for women. Even when other criteria were applied, the association between changes in working status and HGS was confirmed.

**Table 3:** Results of sensitivity analyses using GEE for association between changes in working status and hand-grip strength by different criteria for defining HGS.

	Criteria	Changes in working status	Low hand-grip strength	
			OR	95% CI
Men	EWGSOP2^a^	Working → working	1.00	
		Working → non-working	1.50	(1.28–1.75)
		Non-working → working	0.97	(0.76–1.23)
		Non-working → non-working	1.46	(1.28–1.67)
	KLoSA^b^	Working → working	1.00	
		Working → non-working	1.54	(1.32–1.81)
		Non-working → working	0.91	(0.71–1.16)
		Non-working → non-working	1.48	(1.29–1.69)
Women	EWGSOP2^a^	Working → working	1.00	
		Working → non-working	1.57	(1.34–1.84)
		Non-working → working	0.90	(0.70–1.16)
		Non-working → non-working	1.48	(1.29–1.69)
	KLoSA^b^	Working → working		
		Working → non-working	1.28	(1.06–1.55)
		Non-working → working	1.18	(0.94–1.48)
		Non-working → non-working	1.34	(1.17–1.55)

*GEE* Generalized estimating equation, *OR* odds ratio, *CI* confidence interval, *EWGSOP2* European Working Group on Sarcopenia in Older People2, *KLoSA* Korean Longitudinal Study of Ageing.

ORs were adjusted for other covariates, respectively.

^a^The criteria for low hand-grip strength defined by EWGSOP2 were < 27 kg for men and < 16 kg for women.

^b^The lower 25 percentiles of the KLoSA data were defined as the low hand-grip strength group, and the criteria were < 26.75 kg for men and < 16.25 for women.

## Discussion

In this nationally representative longitudinal panel study, we found an association between changes in working status and HGS among Korean adults aged > 45 years. In particular, those who quit their jobs and continued to be non-working were likelier to have lower HGS than those who continued to work. These results suggest that what affects HGS is the final working status, so it can be interpreted that the risk of low HGS is higher when the final working status is non-working. In addition, there was a difference in the association with HGS between the two groups with changes in working status. Although not statistically significant, the men who changed from non-working to working status were less likely to have reduced HGS. This suggests that if middle-aged and older adults resume economic activity, their muscle strength, including HGS, is expected to improve. Similar to the findings of our longitudinal study, a cross-sectional study proved that there was a higher likelihood of low HGS in unemployed elderly men^30^.

Factors affecting HGS have been identified in previous studies^4^. For example, HGS and its association with cognitive functioning^31–33^, depressive symptoms^34^, and ADL disability^35^ have been studied. Studies have revealed the effects of HGS on health-related outcomes, such as mortality^36,37^ and multimorbidity^38^ in older and ill patients. In addition, the impact of working hours and working conditions on health status or health-related behaviors have already been proven^39–41^. However, few studies have examined the relationship between changes in working status and HGS in community-dwelling adults. As the working status of many people is expected to change due to the outbreak of coronavirus disease 2019 (COVID-19)^42^, the results of this study are thought to be useful as evidence for the development of interventions aimed at promoting strength training among middle-aged and older people.

Studies conducted in Asia generally define HGS weakness according to the AWGS criteria. However, when we redefined the low HGS criteria based on the KLoSA data in the sensitivity analysis, the criteria for both men and women were slightly lower than those obtained with the AWGS criteria. This may mean that the HGS of Koreans is weak compared to Asian standards, further suggesting the lack of strength training in the Korean context. We also suggest that to facilitate advanced research on the effects of sarcopenia on health in the future, Korean criteria should be clearly established.

Our study has certain strengths and limitations. A major strength is that the sample size was relatively large and adequately represented Korean adults; therefore, our results can be generalized to the national level. However, one important limitation of this study is that only absolute HGS was considered when defining the dependent variable. Although there is a concept of relative HGS (RHGS), which is calculated by dividing the BMI by absolute HGS, we did not use RHGS because the height and weight required to calculate BMI were not accurately measured as they were self-reported variables. Instead, we included BMI as a covariate, and a subgroup analysis was performed according to BMI. Second, we adjusted the exercise variable, but we could not confirm the type and duration of the exercise. While it was confirmed that exercise was associated with HGS, it would be useful to conduct additional analyses by supplementing the questionnaire items on the type and duration of exercise. Third, although we attempted to adjust for covariates that may affect HGS, residual confounding effects of unmeasured variables could not be ruled out. For example, we could not adjust for variables related to the type of job and the reasons for the changes in working status. Further studies should complement these factors to introduce more appropriate interventions for the aging population.

## Conclusion

This study identified an association between changes in working status and HGS among middle-aged and older Korean adults. In particular, individuals who quit working or continued to have a non-working status were at a higher risk of low HGS than others. Our findings suggest the need to introduce muscular strength training interventions to improve HGS and prevent sarcopenia in the aging population.

## Supplementary Information

Supplementary Table 1.

## Data Availability

The dataset analyzed in the present study is publicly accessible. Available online: https://survey.keis.or.kr/klosa/klosa01.jsp.

## Abbreviations

AWGS: Asian Working Group for Sarcopenia
EWGSOP: European Working Group on Sarcopenia in Older People
HGS: Hand-grip strength
KLoSA: Korean Longitudinal Study of Aging
