# Supplementary material for: Association between changes in working status and hand-grip strength among Korean middle-aged and older adults: a longitudinal panel study
Source: Sci Rep. 2022 Jul 28;12:12897. doi: 10.1038/s41598-022-16373-2 (PMC9330927; doi:10.1038/s41598-022-16373-2)
Supplement: Supplementary file 1 — Supplementary Table 1. [file 41598_2022_16373_MOESM1_ESM.pdf]

Supplementary Table1. Results of GEE analysis with HGS as a continuous variable

Variables	Hand-grip strength							
	Men				Women			
	β	95% CI			β	95% CI		
**Changes in working status**								
Working→Working	Ref.				Ref.			
Working→Non-working	-0.0385	(-0.050	-	-0.027)	-0.0290	(-0.045	-	-0.013)
Non-working→Working	-0.0097	(-0.026	-	0.006)	-0.0096	(-0.027	-	0.008)
Non-working→Non-working	-0.0442	(-0.053	-	-0.035)	-0.0338	(-0.044	-	-0.024)
**Age**								
45~59 years	Ref.				Ref.			
60~69 years	-0.0509	(-0.059	-	-0.043)	-0.0656	(-0.076	-	-0.056)
70~79 years	-0.1277	(-0.138	-	-0.118)	-0.1492	(-0.161	-	-0.137)
80 years or older	-0.2716	(-0.286	-	-0.257)	-0.2576	(-0.275	-	-0.240)
**Marital status**								
Married	Ref.				Ref.			
Unmarried or Being seperately	-0.0004	(-0.012	-	0.011)	-0.0146	(-0.023	-	-0.006)
**Region**								
Urban	Ref.				Ref.			
Rural	0.0088	(0.003	-	0.015)	0.0173	(0.010	-	0.025)
**Highest level of education**								
Middle school or below	-0.0118	(-0.021	-	-0.003)	-0.0220	(-0.040	-	-0.004)
High school	0.0072	(-0.001	-	0.016)	-0.0083	(-0.026	-	0.010)
College and above	Ref.				Ref.			
**Personal income level**								
Tertile 1 (low)	-0.0306	(-0.040	-	-0.021)	-0.0160	(-0.026	-	-0.006)
Tertile 2	-0.0106	(-0.018	-	-0.003)	-0.0165	(-0.026	-	-0.007)
Tertile 3 (high)	Ref.				Ref.			
**Satisfaction with health status**								
Dissatisfied	-0.0722	(-0.085	-	-0.060)	-0.0864	(-0.101	-	-0.072)
Average	-0.0235	(-0.031	-	-0.017)	-0.0242	(-0.034	-	-0.014)
Satisfied	Ref.				Ref.			
**BMI**[a]								
Underweight	-0.0873	(-0.106	-	-0.069)	-0.0735	(-0.096	-	-0.051)
Normal	Ref.				Ref.			
Overweight	0.0369	(0.030	-	0.044)	0.0297	(0.022	-	0.038)
**ADL Index**[b]								
0 (Normal)	Ref.				Ref.			
1 (Need help)	-0.1988	(-0.236	-	-0.162)	-0.0862	(-0.124	-	-0.048)
**IADL Index**[b]								
0 (Normal)	Ref.				Ref.			
1 (Need help)	0.0452	(0.035	-	0.056)	-0.0589	(-0.079	-	-0.038)
**Cognitive function**[c]								
Normal	Ref.				Ref.			
Impairment	-0.0761	(-0.086	-	-0.066)	-0.0829	(-0.092	-	-0.073)
**Regular physical exercise**								
Yes	Ref.				Ref.			
No	-0.0315	(-0.038	-	-0.025)	-0.0100	(-0.018	-	-0.002)

Abbreviations: GEE, Generalized estimating equation; CI, confidence interval; HGS, hand-grip strength; BMI, Body Mass Index; ADL, Activities of Daily Living; IADL,Instrumental Activities of Daily Living.

[a] Underweight (BMI of less than 18.5kg/$m^{2)}$, Normal (BMI of 18.5-24.9kg/m^2), Overweight (BMI of 25kg/m^2 or over).

[b] 0 point (If they do not need any help), 1 point (If they need some help, or if they need help altogether).

[c] Normal (MMSE score of 24 points or more), Impairment (MMSE score of 23 points or less).
